# Safety, Efficacy, and Effectiveness of Pulsed Field Ablation with Farapulse™ to Treat Atrial Fibrillation: A Prospective Single-center Study

**DOI:** 10.19102/icrm.2026.17096

**Published:** 2026-09-15

**Authors:** Dean Sabayon, Deaa Abu Jazar, Khaled Alabdallah, Ritika Saxena, Ivana Garza, Arun Narayanan, Haidar Al Taii, Dustin Staloch, Hani Jneid

**Affiliations:** 1 Cardiovascular Disease Department, The University of Texas Medical Branch, Galveston, TX, USA

**Keywords:** Atrial fibrillation, Farapulse, pulmonary vein isolation, pulsed field ablation

## Abstract

Pulsed field ablation (PFA) using the Farapulse™ system (Boston Scientific, Marlborough, MA, USA) is a novel, non-thermal ablation technology developed to treat atrial fibrillation (AF) with improved safety and procedural efficiency. We present prospective single-center data on the safety, efficacy, and effectiveness of PFA in a real-world patient population. We enrolled 100 consecutive patients with symptomatic AF undergoing catheter ablation with the Farapulse™ system. Procedural characteristics, clinical outcomes, and acute safety events were evaluated. Freedom from AF recurrence was assessed at 6 months. The cohort had a mean age of 69.2 ± 10.9 years, and 47% were women. Pulmonary vein isolation was achieved in 96% of patients, with additional posterior wall ablation performed in 66%. The median procedure and fluoroscopy times were 42 and 12 min, respectively. No major adverse events occurred. At 6 months, 82% of patients were free from atrial arrhythmia recurrence off anti-arrhythmic drugs. PFA with the Farapulse™ system demonstrated high acute efficacy, excellent safety, and promising short-term effectiveness in a real-world population with AF. These findings support the broader adoption of PFA in routine clinical practice.

## Introduction

Atrial fibrillation (AF) is the most common sustained cardiac arrhythmia worldwide and is associated with an increased risk of thromboembolic events, heart failure, and mortality. Catheter ablation has become an established therapy for symptomatic AF, with pulmonary vein isolation (PVI) serving as the cornerstone of the procedure due to the central role of pulmonary vein triggers in the initiation and maintenance of AF. Contemporary clinical guidelines and expert consensus documents support catheter ablation as an effective rhythm-control strategy for appropriately selected patients with AF.^1–3^

Conventional ablation techniques, including radiofrequency and cryoballoon ablation, rely primarily on thermal energy to achieve myocardial tissue injury. While these approaches have demonstrated efficacy, they may be associated with collateral injury to adjacent structures such as the esophagus, phrenic nerve, and pulmonary veins.^4,5^

Pulsed field ablation (PFA) is an emerging energy modality that uses high-voltage electrical fields to induce myocardial cell death through irreversible electroporation. This process creates nanopores in the cell membrane, leading to loss of cellular homeostasis and subsequent cell death.^6,7^ Irreversible electroporation preferentially affects myocardial tissue while relatively sparing surrounding structures such as the esophagus, phrenic nerve, and pulmonary veins. Although electroporation is the primary mechanism of action, limited thermal effects may occur. Preclinical and early clinical studies have demonstrated the myocardial selectivity of electroporation and suggested a favorable safety profile while maintaining effective PVI.^8–10^

The Farapulse™ system (Boston Scientific, Marlborough, MA, USA) represents one of the first commercially available PFA platforms to be adopted in clinical practice. In this study, we report prospective real-world data from a single high-volume center evaluating procedural outcomes, safety, and 6-month effectiveness of PFA-guided ablation using the Farapulse™ system.

## Methods

### Study population

We prospectively enrolled 100 consecutive patients with symptomatic paroxysmal or persistent AF undergoing ablation using the Farapulse™ PFA system. Procedures were performed by three experienced electrophysiology operators at our center. Data were collected on baseline demographics, comorbidities, echocardiographic characteristics, procedural metrics, safety outcomes, and post-procedural arrhythmia recurrence **(Tables 1–3)**.

**Table 1:** Patient Characteristics

Parameter	Value
Age (mean ± SD), years	69.2 ± 10.9
Female sex	47 (47%)
AF type	
Paroxysmal	51 (52.6%)
Persistent	39 (40.2%)
Long-standing persistent	7 (7.2%)
Medical history	
Hypertension	85 (85%)
Hyperlipidemia	63 (63%)
Diabetes	23 (23%)
Coronary artery disease	19 (19%)
Congestive heart failure	42 (42%)
Stroke/TIA	7 (7%)
Valve dysfunction	2 (2%)
Obstructive sleep apnea	33 (33%)
Echocardiographic features	
LVEF >50%	73 (76%)
LVEF 40%–50%	11 (11.5%)
LVEF <40%	12 (12.5%)
LAVI <34 mL/m^2^ (normal)	39 (41.5%)
LAVI 35–41 mL/m^2^ (mild)	24 (25.5%)
LAVI 42–47 mL/m^2^ (moderate)	20 (23.4%)
LAVI >48 mL/m^2^ (severe)	9 (9.6%)
Prior AF ablation	20 (20%)
Prior DCCV	39 (39%)

*Abbreviations:* AF, atrial fibrillation; DCCV, direct current cardioversion; LAVI, left atrial volume index; LVEF, left ventricular ejection fraction; SD, standard deviation; TIA, transient ischemic attack.

**Table 2:** Procedural Characteristics

Characteristic	Value
General anesthesia	95 (95%)
Monitored anesthesia care	5 (5%)
PVI	96 (96%)
Posterior wall ablation	66 (66%)
Anterior wall ablation	1 (1%)
SVC ablation	1 (1%)
Other ablations	6 (6%)
Median procedure time (range), min	42 (19–181)
Median fluoroscopy time (range), min	12 (5–29)
Time from access to transseptal puncture (range), min	8 (2–30)
Time from access to Farawave removal (range), min	33 (28–43)
Lesions delivered	
Median PVI lesions (range)	42 (0–66)
Median PWI lesions (range)	12 (0–29)
Anterior wall lesions	4 (1 patient)
SVC lesions	5 (1 patient)

*Abbreviations:* PVI, pulmonary vein isolation; PWI, perfusion-weighted imaging; SVC, superior vena cava.

**Table 3:** Safety Outcomes

Adverse Events	n (%)
Minor adverse events	
Hematoma/access site bleeding	5 (5%)
Small pericardial effusion (no intervention)	6 (6%)
Major adverse events	
Stroke	0 (0%)
Cardiac tamponade	0 (0%)
Myocardial infarction	0 (0%)
Coronary spasm	0 (0%)

This study was approved by an institutional review board (human studies committee or ethics committee or animal care and use committee). Verbal and written consent was obtained as per University of Texas Medical Branch protocol.

### Effectiveness outcomes

*Follow-up protocol.* All patients underwent standardized follow-up, including clinical evaluations, 12-lead electrocardiography, and 24-h Holter monitoring at 3 and 6 months post-ablation. Additional monitoring with event recorders was used in patients reporting symptoms suggestive of arrhythmia recurrence. Anti-arrhythmic drug therapy was discontinued post-ablation at the discretion of the treating electrophysiologist.

At 6-month follow-up, 82% (82/100) of patients remained free from any documented recurrence of atrial arrhythmias—including AF, atrial flutter, or atrial tachycardia lasting >30 s—off anti-arrhythmic medications. Arrhythmia recurrence was observed in 18 patients, including seven with paroxysmal AF, eight with persistent AF, and three with long-standing persistent AF.

Most recurrences occurred during the 3-month blanking period; however, 12 patients experienced late recurrences (>90 days post-ablation). No patients developed pro-arrhythmic complications such as organized atrial flutter attributable to ablation lesions.

Within the first 3 months, 16.2% (16/99) of patients presented to the emergency department or were hospitalized for PFA-related complications or recurrent AF. Among the 12 hospitalizations with documented reasons, nine (75%) were due to AF and three (25%) were due to pericarditis; there were no cases of pericardial tamponade or stroke. Cardioversion was performed in 8.2% (8/97) of patients within 3 months.

Left ventricular ejection fraction (LVEF) at 3 months was available in 41 patients. Among these, 39.0% had an LVEF of 55%–60%, 19.5% had that of 50%–55%, and 12.2% had that of 60%–65%, respectively. Reduced LVEF was present in a minority of patients, with 7.3% having an LVEF of 20%–25%. Data on LVEF were missing in 59% of patients.

At 6 months, 7.4% (7/95) of patients were hospitalized for cardiovascular events, including AF (n = 3, 42.9%) and heart failure exacerbation (n = 4, 57.1%), with no hospitalizations for stroke. At 12 months, 5.7% (4/70) of patients were hospitalized, all due to heart failure exacerbation. No stroke or AF-related hospitalizations were reported during this period.

**Figure 1:**
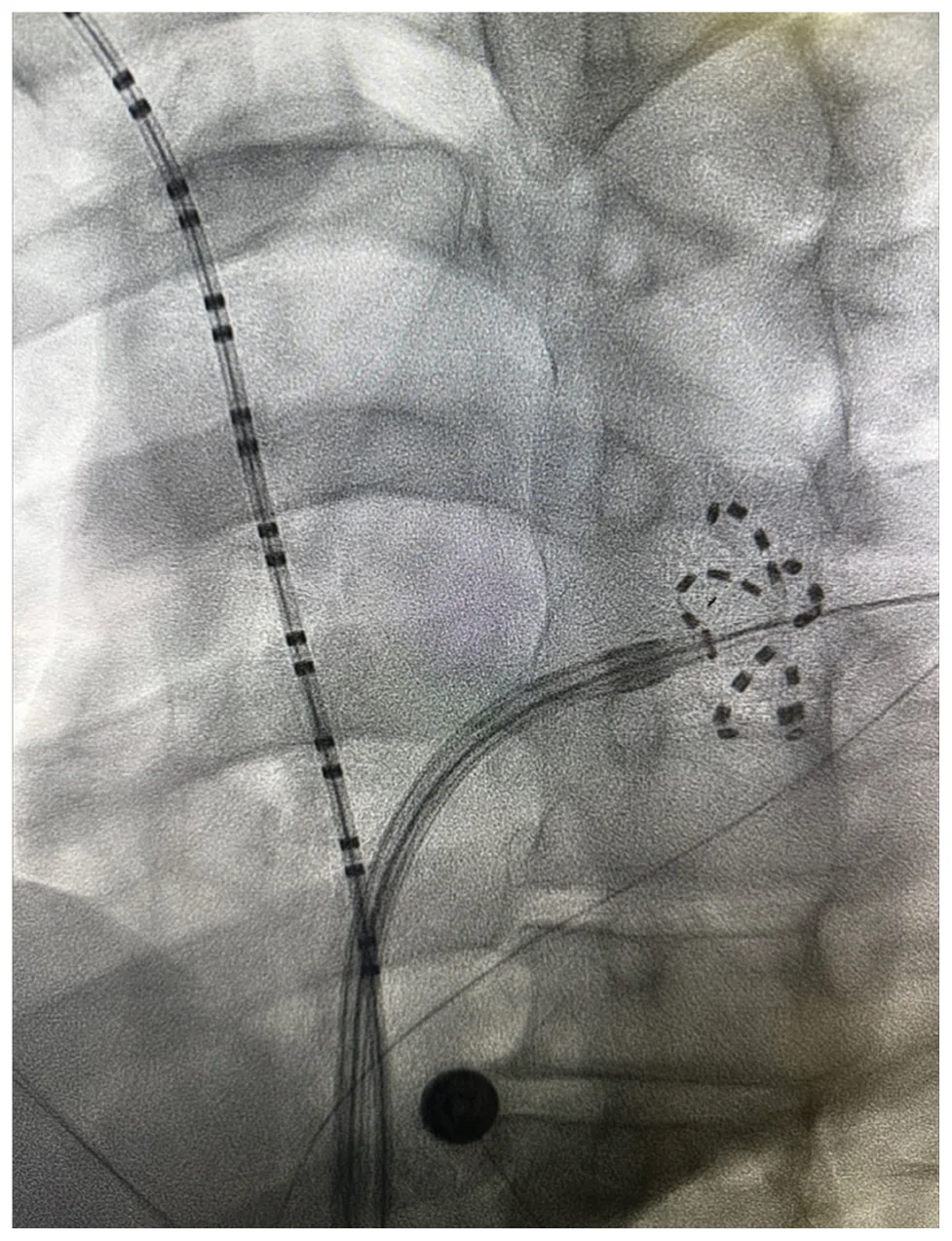
Fluoroscopic imaging demonstrating the position of the Farapulse™ catheter in flower configuration in the left inferior pulmonary vein.

**Figure 2:**
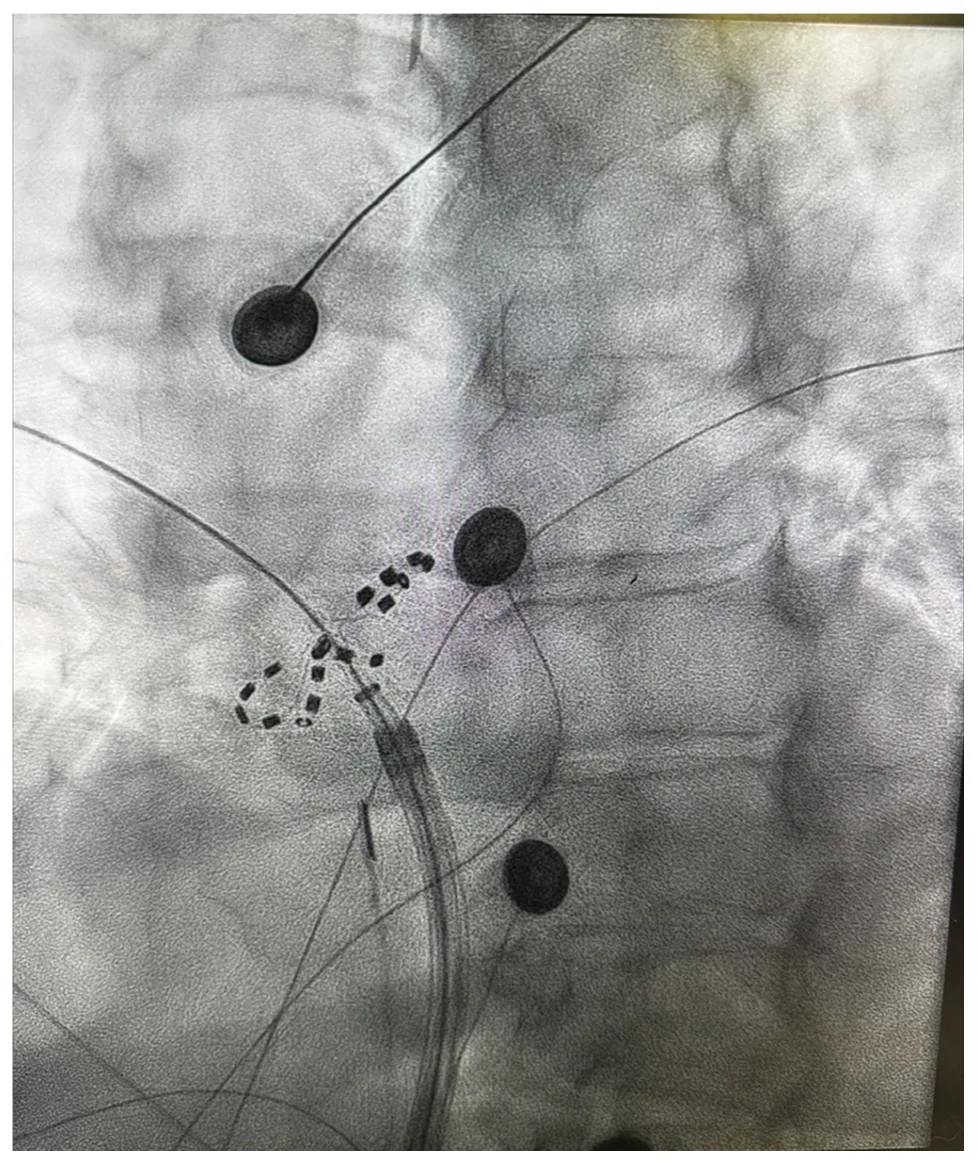
Fluoroscopic imaging demonstrating Farapulse™ catheter position in the right upper pulmonary vein.

**Figure 3:**
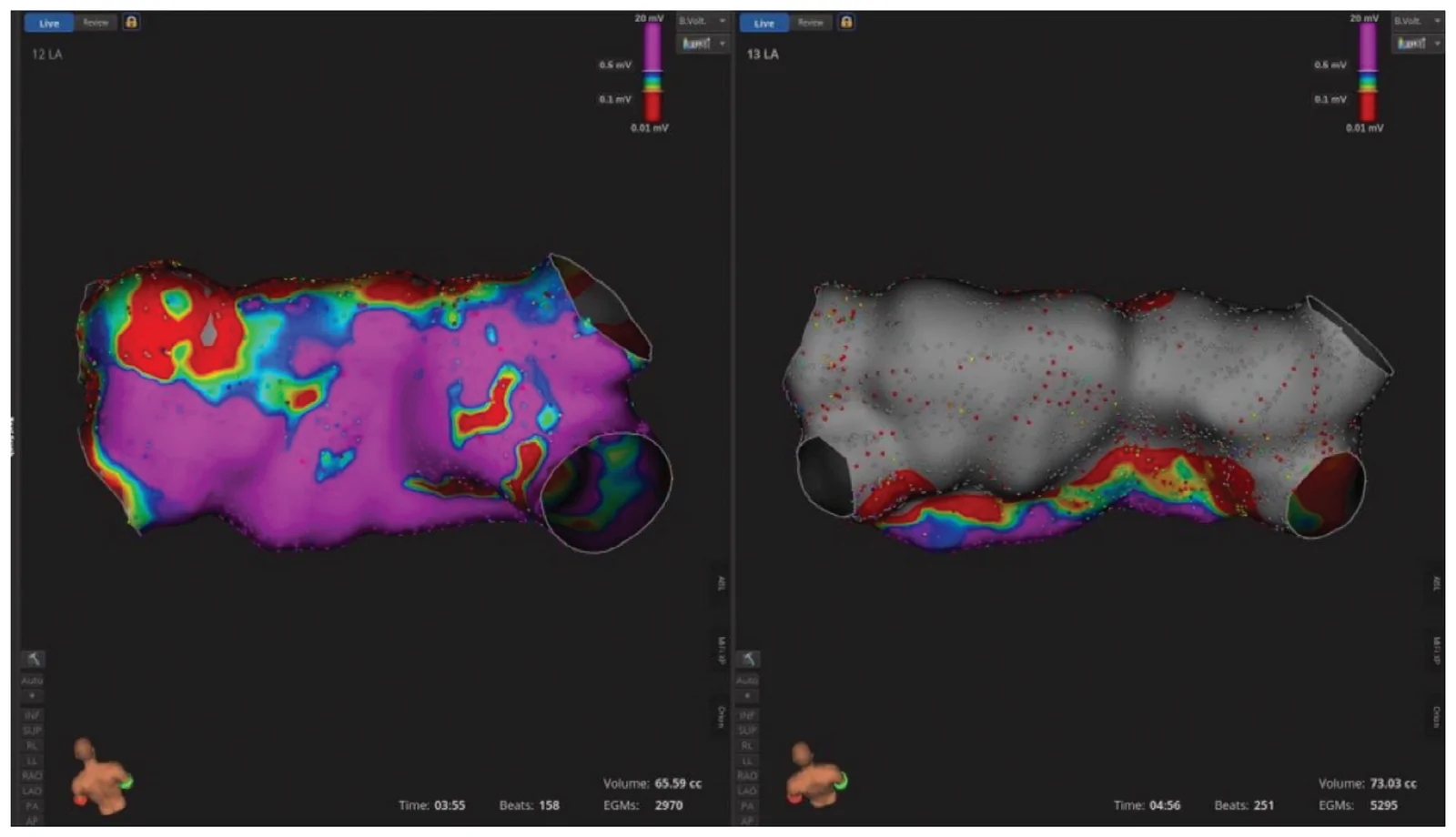
Three-dimensional voltage maps of the left atrium before and after pulsed field ablation demonstrating pulmonary vein and posterior wall isolation.

## Discussion

This study confirms that PFA using the Farapulse™ system is a highly effective and safe method for treating a broad spectrum of patients with AF. Our 6-month freedom from atrial arrhythmia of 82% without anti-arrhythmic medications is consistent with or superior to contemporary thermal ablation techniques, particularly given the inclusion of patients with persistent and long-standing AF.

Posterior wall ablation was performed in a subset of patients with both paroxysmal and persistent AF. In patients with persistent AF, posterior wall ablation was performed at the operator’s discretion. In patients with paroxysmal AF, posterior wall ablation was performed primarily in the setting of recurrent procedures and was guided by findings from three-dimensional electroanatomic mapping.

Following the procedure, patients were monitored for a short observation period and were routinely discharged on the same day. Anti-arrhythmic medications were discontinued immediately after the ablation procedure as part of the post-ablation management protocol.

Notably, this outcome was achieved with a very low complication rate and short procedure times. The nature of PFA likely contributes to this safety profile by reducing risks of esophageal injury, phrenic nerve damage, and pulmonary vein stenosis.

## Conclusion

In a prospective, single-center cohort, PFA with the Farapulse™ system demonstrated high procedural success, excellent safety, and strong effectiveness at 6 months. These findings support the use of PFA as a front-line ablation approach in patients with AF.
